# Engineering of the Filamentous Fungus *Penicillium chrysogenum* as Cell Factory for Natural Products

**DOI:** 10.3389/fmicb.2018.02768

**Published:** 2018-11-15

**Authors:** Fernando Guzmán-Chávez, Reto D. Zwahlen, Roel A. L. Bovenberg, Arnold J. M. Driessen

**Affiliations:** ^1^Molecular Microbiology, Groningen Biomolecular Sciences and Biotechnology Institute, University of Groningen, Groningen, Netherlands; ^2^Synthetic Biology and Cell Engineering, Groningen Biomolecular Sciences and Biotechnology Institute, University of Groningen, Groningen, Netherlands; ^3^DSM Biotechnology Centre, Delft, Netherlands

**Keywords:** *Penicillium chrysogenum*, natural products, nonribosomal peptides, polyketides, gene activation, biosynthetic gene clusters, cell factory

## Abstract

*Penicillium chrysogenum* (renamed *P. rubens*) is the most studied member of a family of more than 350 *Penicillium* species that constitute the genus. Since the discovery of penicillin by Alexander Fleming, this filamentous fungus is used as a commercial β-lactam antibiotic producer. For several decades, *P. chrysogenum* was subjected to a classical strain improvement (CSI) program to increase penicillin titers. This resulted in a massive increase in the penicillin production capacity, paralleled by the silencing of several other biosynthetic gene clusters (BGCs), causing a reduction in the production of a broad range of BGC encoded natural products (NPs). Several approaches have been used to restore the ability of the penicillin production strains to synthetize the NPs lost during the CSI. Here, we summarize various re-activation mechanisms of BGCs, and how interference with regulation can be used as a strategy to activate or silence BGCs in filamentous fungi. To further emphasize the versatility of *P. chrysogenum* as a fungal production platform for NPs with potential commercial value, protein engineering of biosynthetic enzymes is discussed as a tool to develop *de novo* BGC pathways for new NPs.

## Introduction

**FIGURE 1:**
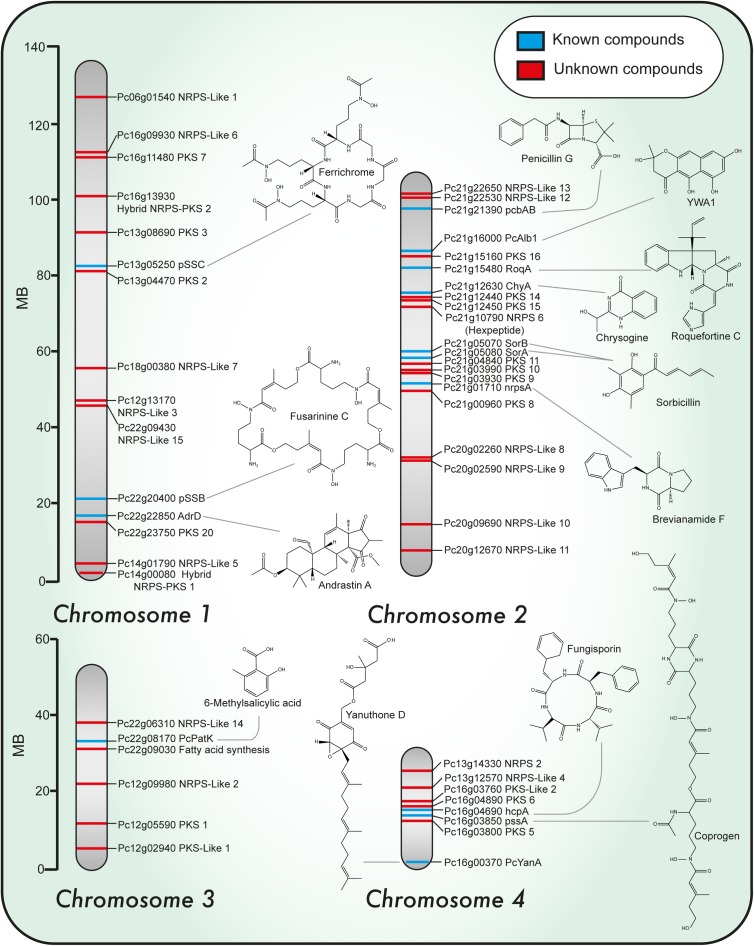
Chromosomal localization of known and predicted PKS and NRPS genes and representative structures of associated secondary metabolites identified in *Penicillium chrysogenum*. Chromosomal localization of PKS and NRPS genes. Blue and red lines indicate known and unknown associated products so far, respectively.

Since the discovery of penicillin by Alexander Fleming produced by the filamentous fungus *Penicillium notatum*, the genus *Penicillium* has been deeply studied for its capacity to produce a wide range of natural products (NPs) (secondary metabolites), many of them with biotechnological and pharmaceutical applications. *P. chrysogenum* (recently renamed as *P. rubens*) is the most relevant member of more than 354 *Penicillium* species that constitute the genus (Nielsen et al., 2017). *Penicillium* is usually found in indoor environments and associated with food spoilage. It is known as an industrial producer of β-lactam antibiotic in particularly penicillin, and current production strains result from several decades of classical strain improvement (CSI) (Gombert et al., 2011; Houbraken et al., 2011). The CSI program began in 1943 with the isolation of *P. chrysogenum* NRRL 1951 capable of growing in submerged cultures. This strain was subjected to a long serial process of mutations induced by 275 nm ultraviolet and X-ray irradiation, nitrogen mustard gas and nitroso-methyl guanidine exposure, single spore selection and selection for loss of pigments, improved growth in large scale industrial fermenters and enhanced levels of penicillin production. CSI programs were developed in several companies (Barreiro et al., 2012), and this has resulted in an increase of penicillin titers by at least three orders of magnitude (van den Berg, 2010). As consequence, numerous genetic modifications were introduced in *P. chrysogenum*. Some have been studied in detail, most notably the amplification of the penicillin biosynthetic clusters and DNA inversions in this region (Fierro et al., 1995, 2006; Barreiro et al., 2012). Although the CSI had a major impact on the production of β-lactams by *P. chrysogenum*, it also affected secondary metabolism in general. Indeed, a proteome analysis performed between *P. chrysogenum* NRRL 1951 and two derived strains (Wisconsin 54-1255 and AS-P-78) showed reduced levels of proteins related to secondary metabolism in the higher penicillin producer strains (Jami et al., 2010). Genome sequencing of *P. chrysogenum* Wisconsin 54-1255 revealed the presence of several secondary metabolite encoding biosynthetic gene clusters (BGCs) in addition to the penicillin cluster, most of which have only be poorly studied and remain to be characterized (Figure 1). The products of the BGCs are either nonribosomal peptides (NRPs), polyketides (PKs) or hybrid molecules.

*Penicillium chrysogenum* produces a broad range of secondary metabolites such as roquefortines, fungisporin (a cyclic hydrophobic tetrapeptide), siderophores, penitric acid, ω-hydroxyemodin, chrysogenin, chrysogine, sesquiterpene PR-toxin and sorbicillinoids, but likely also possesses the ability to produce other compounds not detected before. For most of the identified compounds, the responsible BGCs are unknown. The development of new bioinformatics tools (SMURF, AntiSMASH) (Khaldi et al., 2010; Weber et al., 2015; Blin et al., 2017) and the increase in the number of fungal genomes sequenced to date has opened the possibility to discover new NPs with novel properties (genome mining). The genes involved in the biosynthesis, regulation and transport of secondary metabolites tend to be arranged in the genome in clusters. Importantly, these gene clusters include the core biosynthetic genes which either encode polyketide synthases (PKSs), nonribosomal peptide synthetases (NRPSs) or terpene synthases genes (Smanski et al., 2016). Recently, a global analysis was performed on 24 genomes of *Penicillium* species and this identified 1,317 putative BGCs predominated by two classes based on PKS (467) and NRPS (260) (Nielsen et al., 2017). In *P. chrysogenum* there are 33 core genes in the secondary metabolism that encode 10 NRPS, 20 PKS, 2 hybrid NRPS–PKS, and 1 dimethyl-allyl-tryptophan synthase (van den Berg et al., 2008; Khaldi et al., 2010; Medema et al., 2011; Samol et al., 2016) (Figure 1). A large number of PKS and NRPS enzymes are found also in other *Penicillium* species but only part of these gene clusters are shared, which suggests an unexplored potential of the secondary metabolome even in a single genus.

Here, we summarize the most recent strategies for engineering filamentous fungi with particular attention to *P. chrysogenum*, a promising cell factory of novel products with new application spectra. A brief description of the key biosynthetic enzymes involved in biosynthesis of secondary metabolites in fungi is provided.

## The Building Enzymes of the Natural Products

Nonribosomal peptide synthetases are large, highly structured and complex enzymatic machineries, closely related to other modular enzymes such as PKSs, NRPS–PKS hybrid synthetases and fatty acid synthetases (FASs). They have certain distinct properties in common, the most striking one being their structural division in domains and modules, which is manifested in their shared evolutionary history (Smith and Sherman, 2008). Every enzyme minimally consists of one module, a functionally distinct unit, which allows for the recruitment and subsequent incorporation of a precursor into a growing product. Domains as well as modules are clearly defined and evolutionary exchangeable structures amongst multi-modular enzymes. In the case of PKS and NRPS, this led to the occurrence of a variety of NRPS–PKS hybrids (Du and Shen, 2001; Shen et al., 2005; Li et al., 2010; Nielsen et al., 2016).

### Nonribosomal Peptides (NRPs) and Nonribosomal Peptide Synthetases (NRPSs)

In comparison to most ribosomally derived peptides, NRPs are low molecular weight products. The structural diversity of NRPs is tremendous, mostly due to their chemical complexity. Significantly contributing to this diversity is the fact that NRPS are not only reliant on proteinogenic amino acids, but up until now more than 500 substrates were identified, which serve as NRPS building blocks (Caboche et al., 2008). These molecules are predominantly amino acids, but not exclusively, since fatty acids, carboxylic acids and others substrates have been reported in NRPs (Marahiel et al., 1997). NRP thus represent a diverse group of natural compounds and occur as linear, branched, circular or macrocircular structures (Dang and Sussmuth, 2017; Sussmuth and Mainz, 2017). The natural functions of NRPs are as diverse as their structures. Signaling, communication, metal-ion chelation, host protection are important functions performed by NRPs, though many compounds are not yet fully characterized in this respect. Nevertheless, the characterization of NPs for applied purposes is well developed and led to a vast collection of ground-breaking pharmaceuticals, including antibiotics, anti-fungal agents, immunosuppressants as well as cytostatic drugs (Frisvad et al., 2004; Watanabe et al., 2009; Dang and Sussmuth, 2017).

**FIGURE 2:**
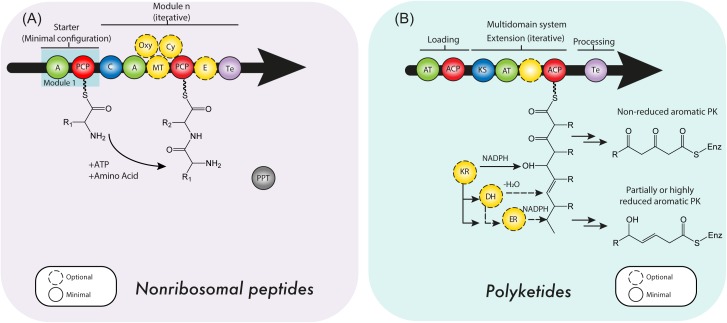
**(A)** Nonribosomal peptide synthetases (NRPSs) and **(B)** polyketide synthase (PKS) minimal domain structure. For details see the text. C, condensation domain; PCP, peptidyl carrier protein; A, adenylation domain; E, epimerase; MT, methyltransferase; PPT, 4’-phosphopantetheine transferase domain; Oxy, oxygenation domain; Cy, cyclization domain; ACP, acyl carrier protein; AT, acyltransferase domain; KS, ketosynthase domain; KR, ketoreductase domain; DH, dehydratase domain; ER, enoyl reductase domain; Te, thioesterase domain.

Structurally, every NRPS module, initiation (1), elongation (n) or termination (1), requires a minimal set of domains (Figure 2A) (Stachelhaus and Marahiel, 1995). The two domains essential to every module are the adenylation domain (A) and the non-catalytic thiolation domain (T). This tandem di-domain enables the specific selection and activation of a given substrate. However, the T-domain must first go through 4′-phosphopantetheinyl transferase (PPTase) and coenzyme A (CoA) dependent activation after expression, by transferring the phosphopantetheine moiety of CoA onto a conserved serine residue, in order to enter the holo state. Also, adenylation domains (A) have accompanying factors, or proteins, called MbtH-like proteins (MLPs) (Quadri et al., 1998; Baltz, 2011). In contrast to PPTases, MLPs are merely interacting with the A-domain, however, they do not have an intrinsic enzymatic activity, but rather a chaperoning function upon binding a distinct part of the A domain (Felnagle et al., 2010; Miller et al., 2016; Schomer and Thomas, 2017). In addition to these domains, any elongation module will require a condensation domain (C), which connects two modules and links up- and downstream activated substrates via a peptide bond. C-domains are stereospecific for both, up- and downstream activated substrates and render the resulting intermediate compound attached to the downstream T-domain. Lastly, the C-terminal termination module essentially requires a thioesterase domain (Te), to catalytically release the covalently bound compound of the NRPS, returning the NRPS complex to the ground state for another reaction cycle. In addition to these essential domains, we can distinguish a series of additional domains, performing epimerization, halogenation, cyclization, macrocyclization, multimerization or methylation (Ansari et al., 2008; Horsman et al., 2016; Bloudoff and Schmeing, 2017).

**FIGURE 3:**
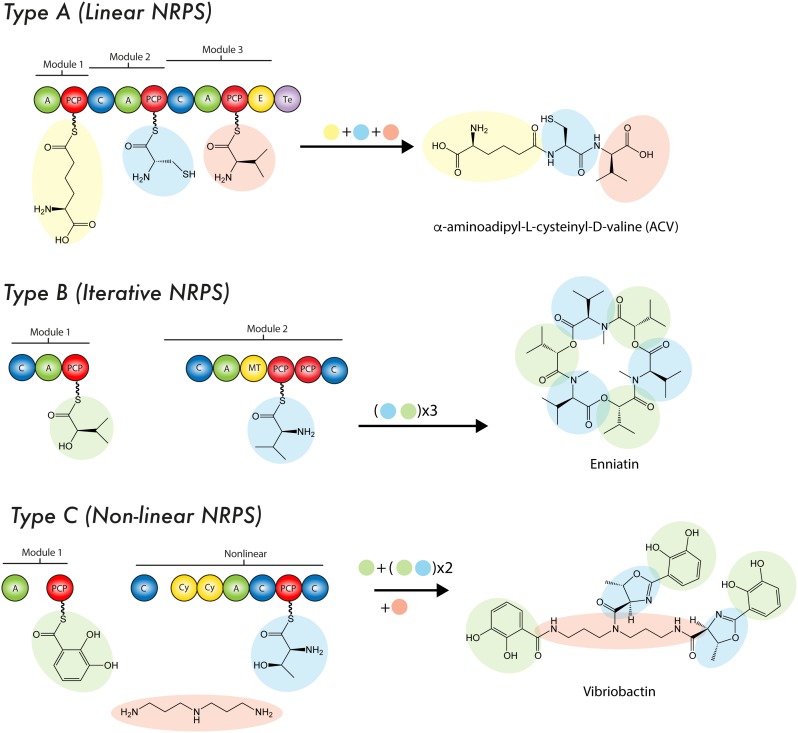
Mode of biosynthesis of nonribosomal peptide synthetases. For details see the text. C, condensation domain; PCP, peptidyl carrier protein; A, adenylation domain; E, epimerase; MT, methyltransferase; Te, thioesterase domain; Cy, cyclization domain.

Theses enzymatic machineries can be classified as *type I NRPS* when the modules are arranged on a single protein, while the *type II NRPS* are independent proteins in an transient manner during the NRP synthesis (Sattely et al., 2008; Hur et al., 2012). A NRPS can be as simple as a single modular unit containing three domains, although the most complex and largest structure known contains 15 modules with 46 domains (Wang H. et al., 2014; Bode et al., 2015) yielding a 1.8 MDa protein complex (type I NRPS). Although the size of a NRPS, as well as the modular sequence, limits the setup of the resulting NRP, it is common that NRPS cluster and interact with tailoring enzymes in order to produce products of a higher complexity (Yin and Zabriskie, 2006). To enable such specific interactions, NRPS can contain small stretches of up to 30 amino acids at the C- or N-terminus, which form a rather specific recognition point, thus enabling communication (COM-domain) between multiple NRPS of one cluster (type II NRPS) (Hahn and Stachelhaus, 2006; Dowling et al., 2016). To date three types of NRPS system have been described according to their synthesis mode (or strategy of biosynthesis): Type A (linear), type B (iterative) and type C (non-linear) (Figure 3). The ***type A system*** harbors the typical domain organization A-T(C-A-T)_n-1_-Te/C, where n represents the number of amino acids in the peptide. In this linear NRPS, the order and number of modules correlates with the amino acid sequence in the NRP and thus it is possible to predict the product that will be formed. Usually, in fungal NRPSs the cyclisation reaction is performed by a specialized C domain instead of Te domain. Since each module catalyzes one cycle during the chain elongation of the nascent NRP due to its specific activity, this system is considered analogous to type I PKS. In fungi, the most prominent examples of this type of NRPS are ACV synthetases (β-lactams), cyclosporin synthetases (Cyclosporin A) and peptaibol synthetases (peptaboils, a class of antibiotics with a high content of α-aminoisobutyric acid) (Wiest et al., 2002; Tang et al., 2007; Felnagle et al., 2008; Eisfeld, 2009). The ***type B system*** is characterized to employ all their modules or domains more than once during the synthesis of a single NRP, which enables the assembling of peptide chains that contain repeated sequences along the structure (Mootz et al., 2002). An example of this mode of synthesis occurs in *Fusarium scirpi* during the biosynthesis of enniatin (antibiotic), which is achieved through the repeated use of two modules. Other examples of type B NRPSs are the siderophore synthetases, which only contain three A domains that catalyze the biosynthesis of ferrichrome (Mootz et al., 2002; Eisfeld, 2009). In ***type C system***, the non-linear NRPSs have at least one domain conformation that deviate from (C-A-T)_n-1_ organization contained in linear NRPSs. Likewise, in these synthetases the module arrangement does not correspond to the amino acid sequence in the NRP. Unlike type A NRPS, in type C NRPSs the non-linear peptide is produced by a branch-point synthase and contains uncommon cyclization patterns. Another important difference is that non-linear NRPSs can incorporate small soluble structures, such as amines into the rising NRP through specialized C domains (Tang et al., 2007; Felnagle et al., 2008; Hur et al., 2012). Capreomycin, bleomycin and vibriobactin are examples of NRPs produced by this type of synthetases (Felnagle et al., 2008). In continuation, a brief description of the main NRPS domains features is provided.

#### Adenylation (A) and Thiolation (T) Domains

Any NRPS module minimally consists of an A- and T-domain (or peptidyl carrier protein, PCP), enabling single module functionality and multi-modular functionality upon addition of C domains (Linne and Marahiel, 2000; Bergendahl et al., 2002; Felnagle et al., 2008; Kittilä et al., 2016; Bloudoff and Schmeing, 2017). They are often referred to as “gatekeeper” domains, as there is no subsequent product formation without prior adenylation and thioesterification of a substrate (Sun et al., 2014). The two core functions of the A-domain are characterized first, through the hydrolysis of ATP or adenylation, allowing an AMP-substrate conjugate to be formed, which is subsequently transferred to the free thiol group of the 4′-phosphopantetheinyl-moiety (Ppant), which is anchored to a conserved serine residue in the downstream T-domain (Ku et al., 1997; Weber and Marahiel, 2001; Neville et al., 2005).

#### Condensation Domains (C)

C-domains are approximately 450 residue NRPS domains, representing a highly versatile class of NRPS domains. Any NRPS composed of more than one module must consequently contain at least one C-domain. However, also single modular NRPS may contain C-domains, especially if they cooperate with other NRPS. Essentially, the primary target of a C-domain is the condensation of the up- and downstream activated substrates through a nucleophilic attack, mainly leading to the formation of an n-peptide linked via a peptide bond. Nonetheless, several residues of the C-domain may have the intrinsic potential to fulfill multiple functions (Balibar et al., 2005; Teruya et al., 2012; Haslinger et al., 2015).

#### Epimerization Domains (E)

The E-domains are among the most abundant modification domains intrinsic to NRPS. In contrast to the structurally similar C domains they are responsible for the site specific epimerization of a substrate, predominantly performing this function after peptide bond formation has occurred (Bloudoff and Schmeing, 2017).

#### Thioesterase Domains (Te)

The thiotemplate based enzymatic systems rely on a catalytic activity in order to remove a product or product-scaffold of the primary enzyme. Therefore, most NRPS contain a domain on their C-terminus responsible for precisely this purpose, the thioesterase domain (Te). Te-domains are a common commodity in single and multi-modular NRPS, although, in multi-NRPS systems only the terminal NRPS contains this domain (Horsman et al., 2016). Additionally, this domain harbors a quality control activity (proofreading) to verify the correct configuration of the nascent peptide (Martín and Liras, 2017).

#### Intrinsic Product Modifying Domains

In addition to the C-domain related epimerization domain, discussed previously, there are cyclization (Cy), oxygenation (Oxy) as well as methyl-transferase (MT). These domains have been characterized to the extent of classifying their functions, although, especially Cy- and Oxy-domains may occur as a singular bi-functional unit or in a serial manner, respectively (Walsh, 2016). Cy- and Oxy-domains, specifically replace the classic function of C-domains, omitting amino acid condensation through peptide bond formation, resulting in thiazoline, oxazoline or methyloxazoline structures (Sundaram and Hertweck, 2016; Walsh, 2016). Those reactions predominantly occur in siderophore producing NRPS and rely on the presence of serine, threonine and cysteine residues (Patel et al., 2003; Kelly et al., 2005). Also MT-domains follow the common di-sub-domain structural patterning, which is also seen in A-, C-, E-, and Cy-domains. Fundamentally, MT-domains, however, are more restricted in their function, which covers the transfer of methyl-groups from *S*-adenosylmethionine to N (N-MT), C (C-MT), O (O-MT) or for certain residues S (S-MT) atoms resolving around the amino acids C_α_ carbon (Miller et al., 2003) and in case of S-MT C_β_, respectively (Al-Mestarihi et al., 2014).

**FIGURE 4:**
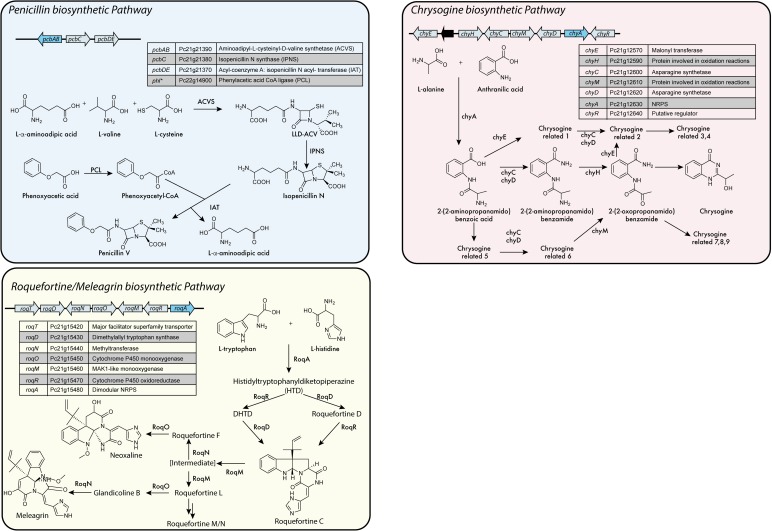
NRPS based biosynthetic pathways in *P. chrysogenum.*^∗^Is not part of the biosynthetic gene cluster (BGC). Adapted from Harris et al. (2009), Bartoszewska et al. (2011), García-Estrada et al. (2011), Weber et al. (2012c), Ali et al. (2013), Ozcengiz and Demain (2013), Ries et al. (2013), and Viggiano et al. (2017).

In *P. chrysogenum* 10 NRPS have been identified (Table 1) (van den Berg et al., 2008; Medema et al., 2011; Samol et al., 2016), of which only two have no attributed function. In this fungus, next to fungisporin (Figure 1) which is a cyclic hydrophobic tetrapeptide generated by a singular NRPS, three biosynthetic pathways involving a NRPS have been described in detail: penicillins, roquefortine/meleagrin, fungisporin and chrysogine (Figure 4).

**Table 1:** Nonribosomal peptide synthetases (NRPSs) in *P. chrysogenum* and known associated products.

Gene ID	Gene name	Protein	Domain organization	Product/Pathway
*Pc13g05250*	*pssC*	Siderophore synthetase	A_1_TCA_2_TCTCA_3_TCTCT	Siderophore
*Pc13g14330*	*–*	Tetrapeptide synthetase	CA_1_TECA_2_TCA_3_TCA_3_TCA_4_TC	–
*Pc16g03850*	*pssA^∗^*	Siderophore synthetase	ATCTA	Coprogen
*Pc16g04690*	*hcpA*	Cyclic tetrapeptide synthetase	A_1_TECA_2_A_3_TCA_4_TECTCT	Fungisporin
*Pc21g01710*	*nrpsA*	Dipeptide synthetase	A_1_TCA_2_T	Brevianamide F
*Pc21g10790*	*–*	Hexapeptide synthetase	A_1_TCA_2_TCA_3_TECA_4_TCA_5_TCA_6_TC	–
*Pc21g12630*	*chyA*	2-Aminobenzamide synthetase	A_1_TCA_2_TC	Chrysogine
*Pc21g15480*	*roqA*	Histidyl-tryptophanyldiketo- piperazine synthetase	A_1_TCA_2_TC	Roquefortine/Meleagrin
*Pc21g21390*	*pcbAB*	α-Aminoadipyl-cysteinyl-valine synthetase	A_1_TCA_2_TCA_3_TEte	β-lactams
*Pc22g20400*	*pssB*	Siderophore synthetase	ATCTC	Fusarinines


*A, adenylation; T, thiolation; E, epimerization; te, thioesterase; C, condensation. ^∗^Point mutations present in *nrps* genes of industrial *P. chrysogenum* strains subject to CSI program. Modified from Salo et al. (2015), Samol et al. (2016), and Guzmán-Chávez et al. (2018).*

### Polyketides and Polyketide Synthase

Polyketides were already discovered in 1883 by James Collie, but the interest in these compounds (enzymes) was revived only as late as the 1950s by the work of Arthur Birch on the aromatic polyketide-6-methyl salicylic acid from *P. patulum*. These molecules are a class of NPs, that display different types of biological activities such as antibiotic (erythromycin A), antifungal (amphotericin B), immunosuppressant (rapamycin), antitumor (geldanmycin) and hypolipidemic (lovastatin) (Nair et al., 2012; Jenner, 2016; Weissman, 2016). Their assembly process is similar to that in the fatty acid biosynthesis, the main difference is the optional full reduction of the β-carbon in the PK biosynthesis. The group of enzymes that catalyzes the biosynthesis of PKs is referred to as PKSs (Keller et al., 2005; Caffrey, 2012).

In addition to the NRPSs, PKSs are the main enzymes that build the structural scaffold of a wide range of secondary metabolites and NPs in plants, bacteria, insects and fungi (Brakhage, 2012; Nair et al., 2012). Usually, these enzymes are encoded by genes that are grouped into clusters, that also specify genes encoding tailoring enzymes (oxygenases, oxidoreductases, reductases, dehydrogenases, and transferases), that further modify the scaffold produced by the PKS into a final product (Brakhage, 2012; Lim et al., 2012). PKSs are multimodular and multidomain enzymes that use a specific acyl-coenzyme A (acyl-CoA; usually malonyl-CoA or methylmalonyl-CoA) as building block, and subsequently catalyze a decarboxylative Claisen-type condensation of ketide units. The basic structural architecture consists of an acyl carrier protein (ACP), a ketosynthase (KS) and an acyltransferase (AT) domain. These combined domains extent a linear intermediate by two carbon atoms. An optional set of domains (dehydratase (DH), ketoreductase (KR), enoyl reductase (ER) and thioesterase (TE) may provide further modifications of the linear intermediate (Staunton and Weissman, 2001; Brakhage, 2012; Nair et al., 2012; Dutta et al., 2014).

**FIGURE 5:**
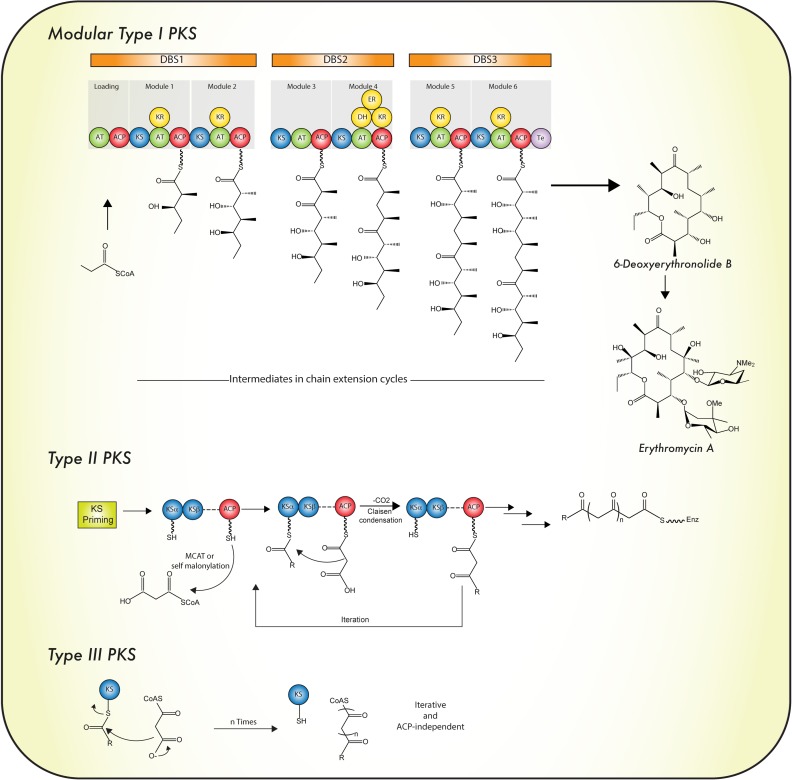
Mode of biosynthesis of polyketide synthases. For details see the text. Abbreviations are as in the legend to Figure 2. Iterative type I PKS is depicted in Figure 2B.

According to their protein architecture and mode of action, PKS enzymes are classified into types I, II, and III (Figure 5). ***Type I PKSs*** are mainly found in bacteria and fungi. These multidomain proteins can be further subdivided in two categories: *modular* and *iterative* (Nair et al., 2012) *Modular type I PKSs* or *non-iterative PKSs* are unique for bacteria and are characterized by presenting a sequence (or set) of modules, each constituted with a set of specific catalytic domains. In consequence, the number of precursors fused in the PK is equivalent to the number of modules which are present (Chan et al., 2009). In contrast, *iterative type I PKSs* use the same catalytic core domains as modular type I PKSs, but the catalytic reaction is repeated to yield the complete PK backbone. A representative example of this type is LovB, that together with LovC (a enoyl reductase) catalyzes around 35 reactions to produce dihydromonacolin L, an intermediate in the lovastatin biosynthesis (Chan et al., 2009; Campbell and Vederas, 2010). Like iterative type I PKS enzymes, fungal PKSs (Figure 2B) are restricted to a single module and the consecutive domains act in sequential order during the synthesis of the complete PK. They are equipped with basic structural domains typically found in PKS enzymes (ACP-KS-AT domains) but may also contain optional units (KR, DH, ER, and Te domains). Depending on the presence or absence of reducing domains, these enzymes can be divided into *highly reducing (HR)*, *non-reducing (NR)* and *partially reducing (PR)* PKS (Figure 2B) (Keller et al., 2005; Crawford and Townsend, 2010; Jenner, 2016).

#### Highly Reducing PKS (HR-PKS)

*Highly reducing PKS (HR-PKS)* produce the linear or cyclic scaffold of some compounds such as fumonisins, T-toxins, solanapyrone E, squalestatin or/and lovastatin (Chiang et al., 2014; Roberts et al., 2017). Usually, they start with a KS domain, followed by an AT, DH and C-Met domain, although the latter does not always follow the DH domain. The ER domain is an optional unit in HR-PKS enzymes, but when the ER is missing, the corresponding region is filled with a polypeptide domain with an unknown function. Furthermore, these enzymes do not contain a product template domain (PT) or N-terminal SAT domain, whereas these special domains are present in NR-PKS enzymes (Cox and Simpson, 2010).

#### Partially Reducing PKS (PR-PKS)

Structurally, these enzymes have a domain architecture that is similar to the mammalian FAS: a N-terminal KS-domain followed by an AT-, DH-, and “*core*”-KR-ACP domain. These enzymes lack an ER domain (Wang L. et al., 2015), and also do not have a Te domain, which suggests an alternative mechanism of product release than hydrolysis. PR-PKS enzymes produce small aromatic molecules such as 6-methylsalicylic acid (MSA), but in most cases the chemical product is unknown (Cox and Simpson, 2009, 2010; Kage et al., 2015).

#### Non-reducing PKS (NR-PKS; Aromatic PKs)

*Non-reducing PKS* (NR-PKS; aromatic PKs) typically, consist of six catalytic domains that are covalently associated and arranged in four components: *loading (SAT), chain extension (KS-MAT-PT-ACP), cyclisation and processing components (TE-CLC)* (Bruegger et al., 2013).

***Type II PKSs*** are unique for bacteria and use a similar iterative mechanism as observed in *iterative type I PKSs*. However, the different catalytic domains are encoded by independent genes. In general, they often constitute a “minimal PKS,” which comprises of two KS units (KS_α_ and KS_β_) and an ACP protein that holds the growing PKS chain. The KS_β_ domain defines the length of the PK chain. The folding pattern of the poly-β-keto intermediates is determined by optional PKS units such as aromatases, ketoreductases, and cyclases. Other tailoring modifications are performed by oxygenases, methyl and glycosyl transferases. Known metabolites synthetized by type II PKSs are tetracyclines, anthracyclines and aureolic acids (Hertweck et al., 2007; Jenner, 2016).***Type III PKSs*** have originally been discovered in plants but are also present in bacteria and fungi. They consist of a single KS domain that catalyzes a defined number of elongations, usually generating small phenols or naphtol rings. The enzyme transfers the acyl group from the CoA to the active site histidine, which is a highly conserved residue. However, the amino acid sequence of the his motif is not similar to those found in KS domains of type I and II PKS enzymes (Shen, 2003; Chan et al., 2009; Bruegger et al., 2014; Jenner, 2016). Importantly, independent of the mechanistic or structural differences, all the PKs synthetized by PKS enzymes follow the same decarboxylative condensation mechanism of the acyl-CoA precursors. However, these precursors should be activated in prior by the ACP domain, in the case of the type I and II PKS enzymes, whereas type III PKS enzymes act independently of ACP domains (Shen, 2003; Hopwood, 2009). Acridones, pyrenes as well as (and) chalcones are some examples of the compounds produced by type III PKS enzymes (Yu et al., 2012). Below, a brief description is provided on the main catalytic features of PKS domains.

#### Acyltransferase Domains (AT)

A main unit during PK biosynthesis is the AT domain that selects the starter unit (malonyl-CoA or methylmalonyl-CoA) before it is transferred to the ACP domain for the chain elongation cycle (Dunn et al., 2013). This process involves two steps, i.e., the acylation and the transfer to the ACP (Jenner, 2016).

#### Acyl Carrier Protein (ACP)

The ACP is an essential cofactor that participates in PK biosynthesis. This protein belongs to a highly conserved carrier family, and consists of 70–100 amino acid residues (Byers and Gong, 2007). To perform the PK biosynthesis, the *holo-ACP* (active) form is generated by the phosphopantetheinyl transferase enzyme (PPTase) through a post-translational modification of ACP whereby a 4′-phosphopantetheine (4′-PP) moiety from CoA is transferred to the conserved serine (Evans et al., 2008; Kapur et al., 2010; Jenner, 2016) resulting in the formation of the Ppant arm. ACP modulates three important events during PK biosynthesis. First, it allows the condensation during chain elongations since it transfers the starter unit from the AT domain to the KS domain. Second, it shuttles the growing chain between the up and downstream domains, as well as to optional PKS domains, probably involving protein–protein recognition between domains. Third, it prevents premature cyclization and enolization of the PK chain (Yadav et al., 2013).

#### Ketosynthase Domains (KS)

The KS is a homodimeric condensing domain that catalyzes the extension of the β-ketoacyl intermediate by a decarboxylative Claisen condensation. This domain contains two active sites which are accessible to the ACP through its flexible Ppant arm, which receives the β-carboxyacyl-CoA extender unit from the AT. At that stage, a thioester bond is formed between the active-site cysteines’ thiol group of the KS and the growing PK. Only when both units are covalently attached onto the module, a decarboxylative Claisen condensation occurs, which involves two conserved his residues. Therefore, mechanistically the KS domain acts at three stages: acylation, decarboxylation and condensation (Chen et al., 2006; Caffrey, 2012; Yadav et al., 2013; Jenner, 2016; Robbins et al., 2016).

#### Ketoreductase Domains (KR)

The KR domain functions as a β-carbon processing unit that belongs to the family of short-chain dehydrogenase/reductases. This domain reduces the β-keto group, that is formed during the condensation process, into a hydroxyl group (a β-hydroxyl intermediate) using NADPH (Keatinge-Clay and Stroud, 2006; Caffrey, 2012). Additionally, some KR domains are equipped with epimerase activity. The epimerizing module has a more open architecture, enabling the catalytic epimerization of methyl groups in acyl-ACP substrates, a reaction that involves the conserved serine and tyrosine residues which are also employed during ketoreduction (Ostrowski et al., 2016c; Bayly and Yadav, 2017).

#### Dehydratase Domains (DH)

The DH domain is usually coupled to B-type KR domains (B-type). This domain catalyzes water elimination (via *syn* or *anti*) at the β-hydroxy acyl chain position thereby producing *trans* double bonds (α,β-unsaturated moieties) (Caffrey, 2012; Bruegger et al., 2014; Jenner, 2016; Bayly and Yadav, 2017).

#### Enoyl Reductase Domains (ER)

The ER domain is an optional tailoring unit involved in the final oxidation state of the growing PK. It reduces α,β-enoyl groups and thereby generates saturated α–β bonds. This reaction involves NAD(P)H as hydride donor in a Michael addition type of mechanism. In the enoyl reduction, the products formed during this reaction have a specific stereochemistry (3R,2R) or (3R,2S) due to the β-carbon attack performed by the pro-4R hydride of NADPH, contrasting the KR domain that utilizes the pro-4S hydride (Chen et al., 2006; Bruegger et al., 2014).

#### Thioesterase Domain (Te)

Termination of PK biosynthesis involves the Te domain, which produces macrolactones via intramolecular cyclization or linear PKs by hydrolysis (Keatinge-Clay, 2012). In both events, an acyl-Te intermediate is formed through the transfer of the PK chain from the last ACP to the active serine on Te domain (Jenner, 2016).

#### Special Domains

In *non-reducing PKS*, the **ACP transacylase (SAT)** domain acts as starter unit that loads the ACP whereupon chain extension is mediated for KS and AT domain. During this process, the malonyl-CoA:ACP transacylase **(MAT) domain** transfers the extension units from malonyl-CoA to the ACP, while the product template **(PT) domain** stabilizes the reactive poly-β-keto intermediates. The processing component acts after the initial assembly when the cyclized or PK intermediate is still attached to the ACP. Final cyclization and release is catalyzed by the Te/Claisen cyclase **(CLC) domain** (Cox and Simpson, 2010; Crawford and Townsend, 2010; Bruegger et al., 2013; Chiang et al., 2014).

**FIGURE 6:**
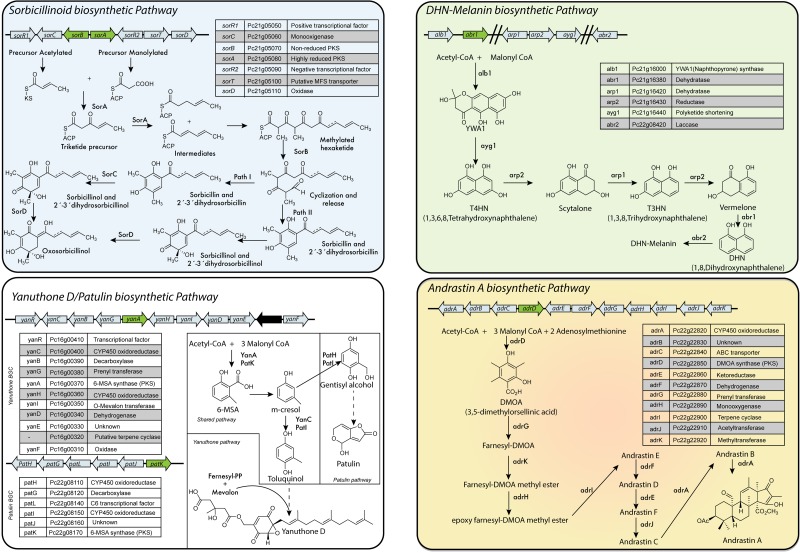
PKS-based biosynthetic pathways in *P. chrysogenum. Sorbicillinoids:* Despite the fact that this cluster is also present in industrial strains of *P. chrysogenum*, they do not produce sorbicillinoids due to a point mutation in the ketosynthase domain of SorA. *Yanuthones/Patulin: P. chrysogenum* only contains a full version of one cluster (yanuthone D BGC), while the second cluster (patulin BGC) is incomplete (Nielsen et al., 2017). The absence of the gene encoding for an isoepoxidon dehydrogenase agrees with the fact that this fungi does not produce patulin (Samol et al., 2016). However, under laboratory conditions, yanuthone D is also not detected in this fungus (Salo, 2016). *DHN-Melanin:* The genes are only partially clustered in the genome of *P. chrysogenum*. *Andrastin A: P. chrysogenum* strains subjected to CSI are not able to produce andrastin A or related compound. Adapted from Staunton and Weissman (2001), Maskey et al. (2005), Cox (2007), Wattanachaisaereekul et al. (2007), Du et al. (2009), Pihet et al. (2009), Crawford and Townsend (2010), Avramovič (2011), Harned and Volp (2011), Gallo et al. (2013), Heinekamp et al. (2013), Matsuda et al. (2013), Salo et al. (2015), Salo et al. (2016), Druzhinina et al. (2016), Meng et al. (2016), Salo (2016), Samol et al. (2016), Guzmán-Chávez et al. (2017), Guzmán-Chávez et al. (2018), Nielsen et al. (2017), and Rojas-Aedo et al. (2017).

In *P. chrysogenum*, 20 PKS genes have been identified (Table 2) (van den Berg et al., 2008; Medema et al., 2011; Samol et al., 2016), but for only six the products are known. To date, in *P. chrysogenum* only four PK-related pathways have been described in detail: sorbicillinoids, MSA-6/yanuthones, DHN-melanin and andrastin A (Figure 6).

**Table 2:** Polyketide synthases in *P. chrysogenum* and (insofar known) their associated products.

Gene ID	Gene name	Protein	Domain organization	Product/Pathway
*Pc12g05590*	*pks1*	–	ks-at-dh-mt-kr-acp	–
*Pc13g04470*	*pks2^∗^*	–	ks-at-dh-mt-er-kr-acp	–
*Pc13g08690*	*pks3*	–	ks-at-dh-mt-er-kr-acp	–
*Pc16g00370*	*yanA*	6-MSA synthase	ks-at-kr-acp	6-MSA/Yanuthones
*Pc16g03800*	*pks5*	–	ks-at-dh-er-kr-acp	–
*Pc16g04890*	*pks6*	–	ks-at-dh-mt-er-kr-acp	–
*Pc16g11480*	*pks7^∗^*	–	ks-at-dh-mt-er-kr-acp	–
*Pc21g00960*	*pks8^∗^*	–	ks-at-dh-mt-er-kr-acp	–
*Pc21g03930*	*pks9*	–	ks-at-dh-mt-er-kr-acp	–
*Pc21g03990*	*pks10*	–	ks-at-dh-er-kr-acp	–
*Pc21g04840*	*pks11*	–	ks-at-dh-er-kr-acp	–
*Pc21g05070*	*sorB^∗^*	Sorbicillin synthase	ks-at-acp-mt-te/red	Sorbicillinoids
*Pc21g05080*	*sorA^∗^*	Sorbicillin synthase	ks-at-dh-mt-er-kr-acp	Sorbicillinoids
*Pc21g12440*	*pks14*	–	ks-at-dh-er-kr-acp	–
*Pc21g12450*	*pks15^∗^*	–	ks-at-acp-te	–
*Pc21g15160*	*pks16*	–	ks-at-dh-mt-er-kr-acp	–
*Pc21g16000*	*alb1^∗^*	YWA1 synthase	ks-at-acp-acp-te	YWA1/DHN-Melanin
*Pc22g08170*	*patK*	6-MSA synthase	ks-at-kr-acp	6-MSA
*Pc22g22850*	*adrD*	DMOA synthase	ks-at-acp-mt-te/red	DMOA/Andrastin A
*Pc22g23750*	*pks20*	–	ks-at-dh-mt-er-kr-acp	–


*ks, ketosynthase; at, acyltransferase; dh, dehydratase; mt, methyltransferase; er, enoyl reductase; kr, ketoreductase; acp, acyl carrier protein; te/red, thioester reductase. ^∗^Point mutations present in *pks* genes of industrial *P. chrysogenum* strains subject to CSI program. Modified from Salo et al. (2015), Samol et al. (2016), and Guzmán-Chávez et al. (2018).*

### Terpenoids Biosynthesis

In addition to NRPs and PKs, terpenoids are another class of NPs that are synthetized by filamentous fungi (Ascomycota) although less abundant as compared to Basidiomycota (Schmidt-Dannert, 2014). Fungal terpenoids or isoprenoids are structurally diverse molecules derived from isoprene units (C5 carbon skeleton): isopentenyl pyrophosphate (IPP) and dimethylallyl pyrophosphate (DMAPP), which are synthetized in the mevalonate pathway from acetyl-CoA (Chiang et al., 2014; Soltani, 2016). The head-to-tail condensation of these C5 units is catalyzed by isoprenyl diphosphate synthases (IDSs), producing isoprenyl diphosphates with 10 (geranyl, GPP), 15 (farnesyl, FPP), and 20 (geranylgeranyl, GGPP) carbons. Eventually, these linear chains of different length are further modified by cyclases, terpene synthases (TPs) and prenyl transferases (PTs), yielding different subclasses of terpenoids (Schmidt-Dannert, 2014; Chen et al., 2016). For instance, monoterpenoids, sesquiterpenoids, diterpenoids, sesterterpenoids, and triterpenoids, which harbor two to six isoprene units, respectively (Soltani, 2016). Terpenoids are oxygenated derivatives of terpenes, which are also derived of isoprene (Stashenko and Martinez, 2017).

In filamentous fungi such as *Aspergillus*, *Penicillium*, *Claviceps*, and *Neosartorya*, ABBA-type PTs are involved in the biosynthesis of a range of toxins (Schmidt-Dannert, 2014). For the synthesis of indole-diterpenoids, IPPS-type PTs transfer GGPP to a indole group, while UbiA-type PTs are involved in the biosynthesis of meroterpenoids, which are hybrid NPs (terpenoids and PKs) (Itoh et al., 2010; Schmidt-Dannert, 2014). In *A. nidulans*, AusN (UbiA-type TPs) converts the product of a NR-PKS (3,5-dimethylorsellinic acid) as part of an earlier step in the dehydroaustinol/austinol biosynthesis pathway (Lo et al., 2012).

Terpene synthases catalyze cyclization reactions forming the carbocation by substrate ionization (class I) or substrate protonation (class II) (Zhou et al., 2012; Meguro et al., 2014). A relevant example of class I TPs are sesquiterpene synthases, which cyclize the FPP to obtain a sesquiterpene scaffold (C15 backbone) (Quin et al., 2014). Recently, the *prx1* to *prx4* gene cluster involved in the biosynthesis of PR-toxin in *P. roqueforti* was cloned and sequenced. This cluster contains the gene *prx2* (*ari1*) that encodes for a aristolochene synthase which forms a sesquiterpene aristolochene derivative (precursor of PR-toxin). Interestingly, an orthologous gene cluster was identified in *P. chrysogenum* (Pc12g06300 to Pc12g06330), as part of BGC of eleven genes, which is also involved in the biosynthesis of PR-toxin (Hidalgo et al., 2014).

## Strategies for Activation of BGCs

**FIGURE 7:**
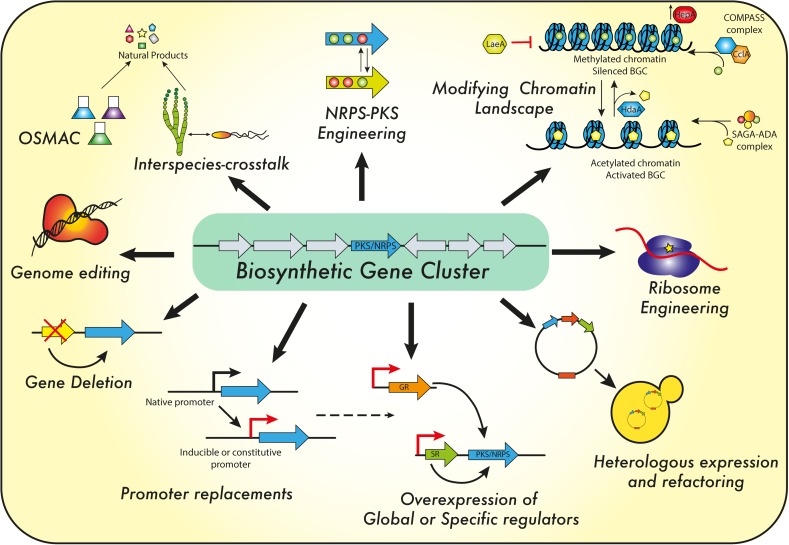
Regulatory targets and strategies to engineer *P. chrysogenum* and other filamentous fungi for secondary metabolite formation. For details see the text.

Natural products represent a broad range of molecules produced by animals, plants and microorganisms. These molecules may display different biological activities (e.g., antiviral, antimicrobial, anti-tumor, immunosuppressive agents) and it is estimated that the majority of these compounds are derived from filamentous fungal sources and from filamentous bacteria belonging to the genus *Streptomyces*. With respect to antibiotics, most of the chemical scaffolds used today were discovered during the golden age of antibiotics discovery (1940–1960s). This was followed by four decades during which hardly any new scaffolds from a natural source were developed (Reen et al., 2015; Smanski et al., 2016; Okada and Seyedsayamdost, 2017). However, there is also a current understanding that only a small fraction of the potential possible molecules has been discovered to this date. This follows from genomic studies revealing large numbers of uncharacterized BGCs, while many of these gene clusters are not expressed (silent or sleeping gene clusters) under laboratory conditions (Brakhage and Schroeckh, 2011). Furthermore, metagenomics studies indicate that the majority of microbes present in the environment have not been cultured nor characterized. Thus, there are many challenges that need to be overcome in order to harness the natural diversity of NPs, to cultivate potential strains under laboratory conditions and to activate the BGCs for expression. To achieve the synthesis of new NPs, three main approaches (Figure 7) were used in recent years, which may be successfully applied in *P. chrysogenum*: manipulation of cultivation conditions, engineering of NRPS and PKS and genetic interference.

### Manipulation of Cultivation Conditions

Under natural conditions, fungi face a variety of biotic and abiotic conditions to survive. The cellular response to the environment involves complex regulatory networks that respond to stimuli such as light, pH, availability of carbon and nitrogen sources, reactive oxygen species, thermal stress, and interspecies-crosstalk (Brakhage, 2012; Reen et al., 2015).

#### OSMAC (One Strain Many Compounds) Approach

This strategy is derived from the observation that changes in the metabolic output of microorganisms can be achieved by alternating the medium composition and other cultivation parameters. It is well known that glucose, ammonium, or phosphate at high concentrations act as repressors of secondary metabolism, whereas iron starvation and nitrogen limitation can stimulate secondary metabolite production. The latter is for instance exploited for the production of terrain by *A. terreus* (Bode et al., 2002; Brakhage and Schroeckh, 2011; Gressler et al., 2015). This strategy can readily be implemented using high-throughput methods, where an array of culture conditions can be screened for new metabolite profiles (Spraker and Keller, 2014). In combination with bioinformatics tools, this strategy can be a powerful tool to investigate the production of new molecules, as exemplified by the discovery of aspoquinolones A–D in *A. nidulans* (Scherlach and Hertweck, 2006). However, despite the fact that the OSMAC approach has led to the discovery of increased numbers of new molecules with antimicrobial activity, some chemical and physical conditions are still missing under the laboratory tested conditions as the activation often concerns a limited number of BGCs (Chiang et al., 2009).

#### Interspecies-Crosstalk

The production of secondary metabolites is a natural strategy that microorganisms have developed to cope with specific environmental conditions and challenges. They serve as intermediary agents to establish a symbiotic association between species or as a weapon against other organism to compete for nutrients and space. These conditions, that are not present in axenic cultures, boost the production of molecules that are constitutively present and/or that are cryptic and normally are not synthetized due to silencing of the respective BGCs (Demain and Fang, 2000; Marmann et al., 2014). The strategy in which different organisms are cultivated together is called “*co-culture*,” which has been successful in several cases yielding new metabolites. *A. fumigatus* produces fumiformamide when co-cultivated with *Streptomyces peucetius*, while co-cultivation of this fungi with *S. rapamycinicus* results in the production of fumicyclines A and B, two novel PKs with antibacterial activity, are examples of the use of this strategy (Netzker et al., 2015; Adnani et al., 2017). Interestingly, the association of two marine organisms, *Emericella* sp. and *Salinispora arenicola*, results in the biosynthesis of emericellamides A and B which are equipped with antibacterial activity (Oh et al., 2007). Also, the interactions between fungi and insects result in the production of volatile secondary metabolites (Rohlfs and Churchill, 2011).

### Engineering of NRPS and PKS

Nonribosomal peptide synthetase and PKS are highly structured and multi-facetted enzymes, containing a tremendous potential for the exploitation of their product scaffold structure for the generation of novel, bioactive compounds. However, due to the complexity of all interactions within these mega enzymes, the elucidation and implementation of engineering strategies is an extremely challenging task. Several strategies have been developed and applied with different degrees of success, though the overall approaches can be grouped as module, domain, sub-domain or site directed, respectively. Owing to their large size, utilization of a random mutagenesis approach proved to be difficult, but other more directed strategies are met with a great success. Nevertheless, all of these strategies have their inherent difficulties, advantages and disadvantages in respect to the complexity and success rate of NRPS/PKS engineering efforts.

#### Subunit, Module, and Domain Swapping

Extensive efforts targeting the active site of A-domains has been a major focus in NRPS engineering. Multiple studies confirmed that the substrate specificity of a NRPS A-domain can be successfully altered, however, at the cost of substantially lowered catalytic velocity (Thirlway et al., 2012; Zhang et al., 2013). Similar successes and limitations were observed when domains were swapped or replaced by synthetic versions (Beer et al., 2014). The most challenging way of obtaining novel NRPS, however, is the swapping or combining of entire modules (Kries, 2016). Domain swapping overall created not only functional parts or domains, but also complete NRPS though with limited success (Beer et al., 2014).

Due to the strict arrangement of NRPS in domains and modules, the possibility of exchanging a unit appears to be the most straight forward approach for altering its intrinsic properties. A series of studies targeting the enzymes linked to the production of daptomycin (Nguyen et al., 2006; Baltz, 2014) elucidated the possibilities and borders of a combinatorial swapping strategy in context of novel compound production. The daptomycin biosynthetic cluster comprises three NRPS containing a total of 13 modules for the incorporation of an equal number of substrates. Different levels of domain and module swap approaches were followed, starting with the exchange of modules 8 and 11 (C-A-T), representing an internal module exchange. The resulting NRPS exhibited the production of novel daptomycin compounds with an inverted amino acid composition at the predicted sites at a near native rate (Nguyen et al., 2006). A similar combinatorial approach has been chosen for altering the PK stereochemistry. The exchange of a R domain with a TE domain in a NR-PKS from *A. niger* produced two alternative NR-PKS that harbor carboxylic acids instead of the aldehydes present in the original products (Yeh et al., 2013; Weissman, 2016). In *Aspergillus*, this rational domain swap has also been used to diversify the native substrates that NR-PKS takes as starter unit to produce new products. This involved exchanging the starter unit ACP transacylase domain in the PKS (Liu et al., 2014). Likewise, an analogous approach was used to produce new hybrids (PK–NRPs) in *A. nidulans* via module swapping of the two PKS–NRPS natural hybrids involved in the syn2 and cytochalasin E pathways from *Magnaporthe oryzae* and *A. clavatus* respectively (Nielsen et al., 2016). Despite the successful use of this strategy in some filamentous fungi, the engineering of NRPS and PKS in *P. chrysogenum* remains unexplored.

### Genetic Interference

Another mechanism to stimulate the expression of silent BGCs in *P. chrysogenum* is by genetic interference, for instance by direct manipulation of the regulatory network related to BGCs expression. The regulation of BGCs is effected at many levels, through specific (or local) and global regulators up to epigenetic regulation involving the modification of the chromatin landscape (Lim et al., 2012; Spraker and Keller, 2014).

#### Global and Specific Regulators

##### Global regulator-based regulation

Pleiotropic transcriptional regulators or global regulators are proteins that respond to environmental signals such as pH, temperature, and N- and C-sources. They provide the link between the production of secondary metabolites and external cues. In fungi, these proteins control the regulation of BGCs that do not contain other regulatory factors. Up to 40% of the known clusters do not encode a local and specific regulator (or obvious regulatory genes). Additionally, global regulators also act on genes that do not belong to secondary metabolism (Brakhage, 2012; Rutledge and Challis, 2015; Fischer et al., 2016). Global regulators that have been reported as key players in the biosynthesis of secondary metabolites are featured below.

###### Velvet complex

This heterotrimeric complex is a conserved regulator present in most of the fungi, except yeast. It consists of at least three proteins: VeA, VelB, and LaeA. Likewise, this complex provides a link between sexual development and secondary metabolism through light regulation (Yin and Keller, 2011; Deepika et al., 2016), since light has an inhibitory effect on VeA expression. The formation of the velvet complex takes place in the nucleus, where the complex VeA–VelB via the α-importin KapA meets the methyltransferase LaeA. It has been hypothesized that the velvet complex acts as a transcriptional factor as it contains a DNA binding fold that resembles the corresponding region of the NF-κB transcription factor of mammals (Sarikaya-Bayram et al., 2015). The role of the velvet complex in secondary metabolism mostly follows from the control that the LaeA protein executes on several BGCs in filamentous fungi. LaeA (loss of aflR expression-A) was identified in 2004 as a global regulator in *Aspergillus*. Deletion of this gene results in the repression of many BGC, such as the one responsible for the production of penicillin, lovastatin, and sterigmatocystin. Overexpression of LaeA causes an opposite phenotype. Interestingly, LaeA is negatively regulated by AflR (Zn_2_Cy_6_ transcriptional factor) in a feed loop mechanism (Bok and Keller, 2004). It has been hypothesized that LaeA acts at different levels, i.e., as a methyltransferase, epigenetically and as a direct member of the velvex complex. Structurally, LaeA has a *S*-adenosyl methionine (SAM)-binding site with a novel *S*-methylmethionine auto-methylation activity, although this activity does not seem to be essential for its function. LaeA is not a DNA-binding protein, but it does affect chromatin modifications. In an *A. nidulans ΔlaeA* strain, high levels of the heterochromatin protein 1 (HepA) are detected and an increase in trimethylation of the H3K9 in the sterigmatocystin cluster. When LaeA is present, the levels of HepA, ClrD (H3K9 methyltransferase) and H3K9me3 decrease while the sterigmatocystin levels are raised. The heterochromatic marks stay until the sterigmatocystin cluster is activated, and apparently LaeA influences the offset of these marks in this particular cluster (Reyes-Dominguez et al., 2010; Brakhage, 2012; Jain and Keller, 2013; Sarikaya-Bayram et al., 2015; Bok and Keller, 2016). Orthologs of LaeA have been discovered in many other filamentous fungi as *Penicillium*, *Fusarium*, *Trichoderma*, *Monascus* spp. and LaeA exhibits positive and negative effects on the synthesis of NPs. For instance, LaeA1 of *F. fujikuroi* positively regulates the production of fusarin C, fumonisins and gibberellins, and represses bikaverin biosynthesis. In *P. chrysogenum*, LaeA controls the biosynthesis of penicillin, pigmentation and sporulation (Keller et al., 2005; Kosalková et al., 2009; Jain and Keller, 2013). In *Trichoderma reesei*, Lae1 positively modulates the expression of cellulases, xylanases, β-glucosidases. Interestingly the stimulation of these genes was not directly influenced by the methylation of H3K4 or H3K9 (Wiemann et al., 2010; Yin and Keller, 2011; Lim et al., 2012; Seiboth et al., 2012; Jain and Keller, 2013).

LaeA is not the only member of the velvet complex that has influence on the regulation of secondary metabolite production. *VeA* of *A. parasiticus* is necessary for the expression of two transcriptional factors of the aflatoxin cluster (AflR and AflJ), which regulate the pathway. In *A. fumigatus*, veA regulates 12e BGCs (Dhingra et al., 2013). This study also revealed that veA modulates the biosynthesis of fumagillin via the regulation of FumR, a transcriptional factor of the fumagillin cluster, which in turn is also regulated by LaeA. Similarly, a transcriptome analysis in *A. flavus* revealed that 28 of 56 BGCs are dependent on *veA*, in particular the aflavarin cluster which is differentially expressed. Likewise, orthologs of veA are also present in other fungi such as in *P. chrysogenum*, *F. oxysporum, Botrytis cinerea*, *F. verticillioides* (Yin and Keller, 2011; Dhingra et al., 2013; Jain and Keller, 2013; Cary et al., 2015). Despite the clear interaction between veA and LaeA in the velvet complex and its influence on secondary metabolism, it is thought that veA may be acting as molecular scaffold of the velvet complex, since it interacts also with three other methyl transferases [LaeA-like methyltransferase F (LlmF), velvet interacting protein C (VipC), and VipC associated protein B (VapB)]. This suggests that veA functions in a supercomplex or in dynamic network control. Taken together, modulation of the velvet complex is a useful tool to activate BGCs (Sarikaya-Bayram et al., 2015) but results are difficult to predict.

###### bZIP transcription factors

Basic leucine zipper (bZIP) transcription factors are highly conserved in the eukaryotes. The dimeric bZIP transcriptional factors play an important role in the cellular responses to the environment. Regarding their structure, they contain a conserved leucine zipper domain and a basic region, which controls the dimerization of the protein and establishes sequence-specific DNA-binding, respectively. Once dimeric, bZIPs target palindromic DNA sequences by two mechanisms: redox and phosphorylation (Amoutzias et al., 2006; Knox and Keller, 2015). In fungi, bZIP proteins have been implicated in multiple metabolic processes, such as in the regulation of development, morphology and in stress responses. Several orthologs of the Yap family bZIPs, which were first described in yeast, have been characterized in *Aspergillus* spp. (AtfA, NapA, Afyap1, Aoyap1, and Apyap1) and these regulators have recently been associated with the production of secondary metabolites in filamentous fungi. In *A. nidulans*, overexpression of *RsmA* (restorer of the secondary metabolism A, Yap-like bZIP) has a compensatory effect on secondary metabolism in a strain in which LaeA and veA are missing. However, these transcription factors also display negative regulation. For instance, an increase in the biosynthesis of aflatoxin and chratoxin has been observed when *yap1* is deleted in *A. parasiticus* and *A. ochraceus* (Yin et al., 2013; Knox and Keller, 2015; Wang X. et al., 2015). MeaB is another bZIP transcriptional factor which was discovered in *A. nidulans*. Its function is associated in nitrogen regulation and has a negative effect on the biosynthesis of aflatoxin in *A. flavus* and bikaverin production in *F. fujikuroi* (Wagner et al., 2010; Amaike et al., 2013).

###### Other global regulators

AreA is a highly conserved transcriptional factor in fungi that belongs to the GATA family and it is characterized by Cys_2_His_2_ zinc finger DNA binding domains. Likewise, it is involved in the repression of nitrogen metabolism when ammonium or glutamine are present. Recently, this transcription factor and its orthologs have been shown to influence secondary metabolism. For instance, *areA* deletion strains of *F. verticillioides* are not able to produce fumonisins on mature maize kernels. In *Acremonium chrysogenum*, the deletion of *areA* resulted in the reduction of cephalosporin because of a reduced expression of the enzymes involved in cephalosporin biosynthesis. Additionally, AreA is a positive regulator of the production of gibberellins, trichothecene deoxynivalenol (DON), fusarielin H, beauvericin and zearalenone (Li et al., 2013; Tudzynski, 2014; Knox and Keller, 2015 and Keller, 2015). The carbon catalytic repressor CreA also influences secondary metabolism. CreA is a Cys_2_His_2_ zinc finger transcription factor that is involved in the repression of genes associated with the use of carbon sources other than glucose (Knox and Keller, 2015). This transcription factor acts by direct competition with activator proteins for specific binding sites (5′-SYGGRG-3′) and by direct interaction with activators (Janus et al., 2008). In *P. chrysogenum* CreA represses penicillin biosynthesis and causes a reduced expression of the *pcbAB* gene that encodes a NRPS involved in this pathway. Mutations in the putative CreA binding site in the *pcbAB* promoter result in enhanced enzyme expression when cells are grown in the presence of glucose (Cepeda-García et al., 2014). In contrast, mutations in the CreA binding sites of the *ipnA* promoter (*pcbC* in other species) of *A. nidulans* revealed that in this organism repression of penicillin biosynthesis by glucose is independent of CreA (Knox and Keller, 2015). CreA has been implicated in the variable metabolite profiles when fungi are grown in the presence of different carbon sources (Yu and Keller, 2005). Recently, the xylanase promoter binding protein (Xpp1) of *Trichoderma reesei* was used as a reporter to fulfill a dual role in the regulation of primary and secondary metabolism. Xpp1 is an activator of primary metabolism, while its deletion boosts the production of secondary metabolites, including sorbicillinoids (Derntl et al., 2017b). Another Cys_2_His_2_ zinc finger transcription factor conserved in fungi is PacC, which is involved in pH dependent regulation. Deletion of the ortholog of this gene (BbpacC) in *Beauveria bassiana* resulted in a loss of dipicolinic acid (insecticide compound) and oxalic acid production, compounds that reduce the pH of the medium. However, also production of a yellow pigment was noted. When *A. nidulans* is grown at alkaline pH, PacC modulates the expression of the *acvA (pcbAB)* and *ipnA* of the penicillin BGC, while it acts negatively on the expression of the sterigmatocystin BGC (Deepika et al., 2016; Luo et al., 2017). In filamentous fungi, another global regulatory element is the CCAAT-binding complex (CBC). This complex consists of three proteins (HapB, HapC, and HapE) that respond to redox stimuli and an additional unit HapX, a bZIP protein that interacts with the complex for modulating the iron levels. In *A. nidulans* this complex binds to CAATT motifs, which are present in the penicillin BGC stimulating the expression of the *ipnA* and *aatA* (*penDE*) genes (Bayram and Braus, 2012; Brakhage, 2012). Whereas in *F. verticillioides* the ortholog core of this complex (FvHAP2, FvHAP3, and FvHAP5) is deleted, cells show an altered hyphal morphology, reduction of growth, reduced pathogenesis and a deregulation of secondary metabolism (Ridenour and Bluhm, 2014).

##### Specific regulator-based regulation

In addition to the global regulators, the expression of BGCs can be also modulated by specific regulatory elements, which most of the times are encoded by genes that are part of the same cluster that they regulate. In some cases, such regulators also influence the expression of other BGCs. It is estimated that about 60% of the fungal BGCs contain a gene encoding a potential regulator amidst the gene cluster. With PKS containing BGCs mostly containing a regulator that belongs to the Zn_2_Cys_6_ binuclear cluster domain family (around 90%). With NRPS containing BGCs, the putative transcription factors are more diverse. The Zn_2_Cys_6_ family of transcription factors contain a DNA binding domain (DBD) that has two zinc atoms coordinated by six cysteines. There are three sub regions: a linker, a zinc finger and a dimerization domain. Additional to a DBD, these proteins contain two further functional domains, the acidic region and the regulatory domain. These transcription factors can act as monomers, hetero- and homodimers. They recognize single or multiple trinucleotide sequences, commonly CCG triples, in a symmetric or asymmetrical format. The affinity of the DBD for a given DNA stretch is also determined by the nucleotides surrounding this triplet. The transcriptional activity of these proteins is regulated by phosphorylation, exposing the activation and DNA binding domains for DNA binding (MacPherson et al., 2006; Brakhage, 2012). Some of these regulators have been shown to control the expression of BGCs. For instance, in *F. verticillioides* the disruption of *FUM21* gene, that encodes a Zn_2_Cys_6_ protein, reduces fumonisin production as a result of a downregulation of the BGC (Brown et al., 2007). Interestingly, fumonisin production is also regulated by another Zn_2_Cys_6_ protein that is encoded by a gene located outside of the fumonisin cluster (Flaherty and Woloshuk, 2004). Mlcr is another example of a positive regulator that controls compactin production in *P. citrum* (Abe et al., 2002). AflR is a Zn_2_Cys_6_ protein that regulates the biosynthesis of aflatoxin/sterigmatocystin through binding to a palindromic sequence (5′-TCG(N_5_)GCA) that is found in most of the promoters of this BGC, albeit a second binding sequence has been reported that is associated with the autoregulation mechanism of the expression of *AflR*. The disruption of *AlfR* abolishes the production of aflatoxin/sterigmatocystin. Likewise, some BGCs encode multiple regulatory proteins. Next to the *aflR* gene in the aflatoxin cluster resides the *aflS* (formerly *aflJ*) gene. The corresponding transcription factor binds to AflR to enhance the transcription of early and mid-biosynthetic genes in the aflatoxin pathway (Georgianna and Payne, 2009; Yin and Keller, 2011). In *P. chrysogenum* and *Trichoderma reesei*, the sorbicillin BGC is regulated by two transcriptional factors through a coordinated action (Derntl et al., 2016, 2017a; Guzmán-Chávez et al., 2017). Also, regulation of BGCs via crosstalk has been observed in filamentous fungi. For instance, the alcohol dehydrogenase promoter has been used to induce the expression of putative pathway-specific regulatory gene *(scpR)* in *A. nidulans*, which controls the expression of two pathway associated NRPS genes (*inpA* and *inpB*). Surprisingly, two PKS genes (*afoE* and *afoG*) and one transcriptional activator (*afoA*) belonging to the asperfuranone BGC are also upregulated by ScpR, allowing the production of asperfuranone (Bergmann et al., 2010). For some regulators, no clear phenotype is observed. For instance deletion of the *chyR* gene of the chrysogine BGC in *P. chrysogenum*, has no effect on the expression of the corresponding BGC (Viggiano et al., 2017).

##### Manipulation of regulatory elements as strategies for the activation of BGCs

###### Gene deletion

It is a classical strategy that consists of the abolishment of the expression of a certain gene by its elimination whereupon the impact on the metabolite profile is examined by HPLC or LC-MS. A major limitation of this approach is that it can only be used in BGCs that are not totally silenced under laboratory conditions. Using this strategy, it was possible to elucidate the highly branched biosynthetic pathway for the synthesis of roquefortine as well as the biosynthetic pathways of sorbicillinoids and chrysogine in *P. chrysogenum* (García-Estrada et al., 2011; Ali et al., 2013; Ries et al., 2013; Deepika et al., 2016; Guzmán-Chávez et al., 2017; Viggiano et al., 2017). Likewise, this approach can be used to remove transcriptional repressor genes, as in the case of TetR-like pathway-specific repressor proteins, whose deletion induced the production of gaburedins in *Streptomyces venezuelae* (Rutledge and Challis, 2015). Global regulators, such as LaeA have also been targeted using this strategy (Chiang et al., 2009).

###### Promoter replacement

Another method concerns the replacement of the endogenous promoter of the gene(s) in a BGC by a strong constitutive or inducible promoter. For instance in *A. nidulans* replacement of the native promotor of the *scpR* gene (secondary metabolism cross-pathway regulator) for the inducible promoter of alcohol dehydrogenase AlcA induced the expression of a silent cluster that contained two NRPS genes (*inpA* and *inpB)* and *scpR* itself. Additionally, it also led to the expression of the asperfuranone BGC, which is normally silent (Bergmann et al., 2010; Yin and Keller, 2011; Lim et al., 2012). Recently in *P. chrysogenum* a promising promoter toolbox for bioengineering purposes was developed. This included the analysis of four constitutive promoters from *P. chrysogenum* and six from *A. niger*, which were evaluated using a reporter system and assorted by promoter strength (Polli et al., 2016).

###### Overexpression of a specific or global regulator

This approach is one of the most used strategies to turn on cryptic BGCs, since a change in expression level of a regulator may boost the expression of a whole cluster. Usually, this strategy is applied in combination with the *promoter replacement* approach. Using this strategy, i.e., overexpression of the transcription activator ApdR under control of the alcohol dehydrogenase promoter *alcAp*, it has been possible to induce the expression of a hybrid PKS-NRPS BGC in *A. nidulans.* This resulted in the production of aspyridones A and B (Bergmann et al., 2007). Similarly when the global regulator FfSge, which is associated with vegetative growth of *F. fujikuroi*, is overexpressed, some BGCs are forced to express under these unfavorable conditions (low nitrogen concentrations) leading to the identification of the corresponding products (Michielse et al., 2015).

#### Chromatin-Mediated Regulation

In fungal cells, chromosomal DNA is wrapped in a complex of DNA, histone proteins and RNA called chromatin. This chromatin structure consists of a basic unit called nucleosome, which consists of superhelical DNA (147 base pairs) that binds an octamer of four different core histone proteins (two each of H2A, H2B, H3, and H4) in 1.75 turns. It has been shown that modifications of the chromatin structure (boosts or alters) changes gene expression, amongst other genes involved in the biosynthesis of secondary metabolites. Structurally, chromatin represents an obstacle that complicates access of DNA-binding factors to their corresponding binding regions. According to the compaction level, chromatin can be in a dense (heterochromatin) or relaxed (euchromatin) state. These compaction levels are regulated by post-translational modification of the histone proteins by acetylation, methylation, ubiquitination, ethylation, propylation, butylation, and phosphorylation events. Regions that display low transcriptional activity have been associated with the heterochromatic conformation. In contrast, the euchromatic conformation is present in regions with abundant coding sequences and is usually highly active during transcription. Such regions are also linked with hyper-acetylated nucleosomal histones. Likewise, it has been reported that methylation of H3K9, H3K27, and H4K20 are typical markers of the heterochromatin, while in euchromatin methylation occurs at H3K4 (Brosch et al., 2008; Strauss and Reyes-Dominguez, 2011; Gacek and Strauss, 2012; Spraker and Keller, 2014; Rutledge and Challis, 2015).

##### Histone methylation, acetylation, and sumoylation

As mentioned above, LaeA influences secondary metabolite production through chromatin modification. The methylation state of H3K9 has been correlated with the heterochromatin protein A (HepA), since this protein needs the di- and tri- methylation of H3K9 for binding to chromatin and to form heterochromatin. Deletion of LaeA allows the unobstructed binding of HepA to the *AlfR* promoter, thereby affecting the expression of the sterigmatocystin pathway. The deletion of the methyltransferase encoding c*lrD* and *ezhB* genes in *Epichloe festucae*, that act on H3K9 and H3K27, respectively (in axenic culture), results in the activation of the ergot alkaloids and lolitrem BGCs. These compounds are necessary to establish a symbiotic association with the plant *Lolium perenne*. Compass (complex of proteins associated with Set1) which methylates H3K4 in yeast, also impacts secondary metabolism in filamentous fungi. The deletion of one of its components (*cclA*) in *A. nidulans* allowed the activation of a cryptic BGC and the production of emodin (Palmer and Keller, 2010; Gacek and Strauss, 2012; Chujo and Scott, 2014; Netzker et al., 2015; Deepika et al., 2016). Likewise, in *F. fujikuroi* and *F. graminearum*, the deletion of *cclA* caused the overproduction of secondary metabolites derived from BGCs close to the telomeres, but this seems to relate to a H3K4 methylation independent mechanism (Studt et al., 2017). Other types of histone modification may alter the chromatin landscape, such as acetylation which is a reversible process governed by two antagonist enzymes: histone acetyltransferases (HATs) and deacetylases (HDACs). Active transcription is usually associated with histone acetylation, although recently the deacetylation of histones has been shown to cause activation of genes (Brosch et al., 2008). Usually, histones are acetylated by several complexes with acyltransferase activity, such as Saga/Ada and NuA4. In *A. nidulans* a chromatin immunoprecipitation (ChIP) analysis revealed that GcnE and AdaB, the catalytic subunits of the complex Saga/Ada, are needed for acetylation of histone H3 (Deepika et al., 2016). Indeed, the interaction between *A. nidulans* and *Streptomyces rapamycinicus* can be linked to a GcnE dependent increase in the acetylation of H3K14 that shields the promoters of the orsellinic acid BGC. The Saga/Ada complex is a key player in the induction of the penicillin, terrequinone and sterigmatocystin BGCs (Nutzmann et al., 2011; Brakhage, 2012). In contrast, deletion of *hdaA* (encoding a HDAC) in *A. nidulans* resulted in major changes in the metabolite profile (Rutledge and Challis, 2015). HdaA is a class 2 histone deacetylase involved in the regulation of BGCs that are located near the telomeres, such as the penicillin and sterigmatocystin clusters in *A. nidulans*. Indeed, deletion of the *hdaA* gene results in the increased and early gene expression of these two BGCs, and the production of the corresponding secondary metabolites. In *A. fumigatus*, the *hdaA* gene is involved in growth and production of secondary metabolites, and the deletion of this gene increases the production of many secondary metabolites while it causes a reduction of gliotoxin production. In contrast, HdaA overexpression shows the opposite effect (Shwab et al., 2007; Lee et al., 2009). In *P. chrysogenum* was demonstrated that HdaA (histone deacetylase) mediates the transcriptional crosstalk among sorbicillinoids biosynthesis and other BGCs, since a new compound as detected only under conditions of sorbicillinoids production (Guzmán-Chávez et al., 2018).

Histone deacetylases are ubiquitously distributed in filamentous fungi, and therefore HDAC inhibitors can be used to improve the synthesis of NPs by epigenome manipulation (Shwab et al., 2007; Lee et al., 2009). For instance, the metabolite profile of *Cladosporium cladosporioides* and *A. niger* underwent a significant change when these strains were exposed to suberoylanilide hydroxamic acid (SAHA), a HDAC inhibitor, allowing the detection of two new compounds, cladochrome and nygerone A, respectively (Rutledge and Challis, 2015). An exploratory analysis performed in 12 fungi treated with different types of DNA methyltransferase and histone deacetylase inhibitors, revealed the production of new secondary metabolites but also the elevated amounts of known compounds (Williams et al., 2008). In this respect, the chromatin state can directly influence the binding of transcription factors, and thereby modulate expression (Palmer and Keller, 2010; Macheleidt et al., 2016). It has been hypothesized that histone sumoylation may modulate secondary metabolite production. This process is mediated by a small protein termed SUMO (small ubiquitin-like modifier) that shares structural similarity to the ubiquitin protein. In *A. nidulans*, deletion of the *sumO* gene enhanced the production of asperthecin, whereas synthesis of austinol, dehydroaustinol, and sterigmatocystin was reduced. Although the molecular mechanism still needs to be elucidated, it is thought that sumoylation acts at several levels, such as on epigenetic regulators (COMPASS, Clr4, SAGA/ADA and HDACs) or at the level of transcriptional regulators (Brakhage and Schroeckh, 2011; Spraker and Keller, 2014; Wu and Yu, 2015).

##### Modification of the chromatin landscape to activate BGCs

Many fungal BGCs are located in distal regions of the chromosomes. In these heterochromatin rich regions, transcription of the BGCs can be activated by epigenetic regulation. Therefore, the encoding genes of proteins that influence histone modification are prime targets, although these modifications can also be achieved by chemical treatment (Williams et al., 2008; Brakhage, 2012). A recent study in *P. chrysogenum* showed that the expression of a set of PKS and NRPS encoding genes is induced when an ortholog of a class 2 histone deacetylase (HdaA) is deleted. This allowed for the overproduction of sorbicillinoids, the reduction of chrysogine related metabolites and the detection of a new compound whose origin still unknown (Guzmán-Chávez et al., 2018).

#### Other Targets for Regulation

Secondary metabolites produced by fungi can be toxic to the producer organisms, and often fungi are equipped with detoxification mechanisms. One of these mechanisms is toxin excretion by transporters, which are membrane proteins whose genes often localize to the BGCs. Transporters may belong to different protein families but the major facilitator superfamily (MFS) and ABC superfamily are most commonly encoded by BGCs (Keller, 2015). Since biosynthesis of secondary metabolites may take place in different cell compartments, also intracellular transport may be evident (Kistler and Broz, 2015). Despite their assumed biological importance, the deletion of transporter genes from the BGCs often does not impact secondary metabolite production. For instance, deletion of the *A. parasiticus aflT* gene, that encodes a MFS transporter, does not result in reduced aflatoxin excretion, despite the fact that *aflT* belongs to the aflatoxin BGC and its expression is regulated by a specific transcription factor, AflR, of the pathway. Probably, this protein is redundant, and other transporters may participate in excretion, detoxification or self-defense. In *A. fumigatus*, GliA facilitates the excretion of gliotoxin. Similarly, the *tri12* gene contained in the trichothecene BGC encodes for a membrane protein required for the biosynthesis of trichothecene and virulence of *F. graminearum* on wheat crops (Chang et al., 2004; Menke et al., 2012; Wang D.N. et al., 2014; Keller, 2015). Often, however, the deletion of the transporter gene in BGCs has no effect on production. Possibly, these metabolites are also recognized by other promiscuous transporters, or transporters that are not part of the BGC (Keller, 2015). For example, ZRA1 of *Gibberella zae*, whose gene is not localized to the zearalenona BGC, impacts zearalenone production. However, the expression of the *zra1* gene is regulated by the transcriptional factor ZEB2, whose gene localizes to the corresponding BGC (Lee et al., 2011). Also, the penicillin BGC of *P. chrysogenum* lacks a transporter gene whereas export of penicillin occurs against the concentration gradient, probably through the activity of multiple transporter proteins (van den Berg et al., 2008; Kistler and Broz, 2015). Furthermore, compartmentalization of the biosynthesis of penicillin is well documented requiring transport of penicillin precursors across the membrane of intracellular organelles (Weber et al., 2012a,b).

#### Other Genetic Engineering Strategies for the Activation of BGCs

Several approaches have been used to activate the expression of cryptic BGCs in a targeted manner. Usually, this is achieved by manipulation of pathway-specific regulatory genes, or by replacing endogenous promoters for inducible systems or strong promoters (Rutledge and Challis, 2015). The various approaches are summarized in Figure 7.

##### Manipulating biosynthetic pathways by genome editing

Due to the increasing number of sequenced filamentous fungi, it is necessary to make use of efficient genome editing tools to explore new potential sources of secondary metabolites. For many years, the unique strategy available for the genome edition of *P. chrysogenum* was based on the use of ku70/80 disrupted strains to improve the homologous recombination instead of the Non-Homologous End Joining (NHEJ) pathway (Weber et al., 2012a). This strategy allowed for the generation *P. chrysogenum* strains with high copy numbers of the penicillin cluster, the identification of a biosynthetic branch of the roquefortine cluster and the reactivation of the sorbicillinoid gene cluster (Nijland et al., 2010; García-Estrada et al., 2011; Ali et al., 2013; Ries et al., 2013; Salo et al., 2016; Guzmán-Chávez et al., 2017). Recently, a CRISPR/Cas9 based system was developed for genome modifications in *P. chrysogenum* (Pohl et al., 2016, 2018). This study demonstrated that the deletion of full gene clusters is feasible with minimal cloning efforts, which opens the possibilities to engineer new synthetic pathways and the re-factoring *P. chrysogenum* as platform organism.

##### Ribosome engineering

This approach has been applied for activating silent or poorly expressed BGCs (Ochi and Hosaka, 2013). Basically, this concept is derived from the activation of the actinorhodin BGC in *S. lividans* due to a point mutation in the *rpsL* gene, which encodes for the ribosomal S12 protein (Shima et al., 1996). Another successful examples in the BGCs activation have been reported by modifying the transcription and translation pathways via targeting different ribosomal proteins, RNA polymerases (RNAP) and translation factors (Ochi and Hosaka, 2013). In *P. purpurogenum* G59, a marine derived strain, the insertion of gentamicin resistance after treatment with high concentrations of this antibiotic, altered ribosomal functions of this fungus which allowed for the activation of dormant secondary metabolite gene clusters (Chai et al., 2012).

##### Heterologous expression and refactoring

Due to the broad range of molecular tools available to express heterologous pathways in yeast, several attempts have been undertaken to express NRPS and PKS genes with the remainder of the pathway in yeast (Rutledge and Challis, 2015). A recent study demonstrated that the baker’s yeast *Saccharomyces cerevisiae* can be used as a platform to produce and secrete penicillin when the biosynthetic genes are expressed in this organism (Awan et al., 2016). Although the first step was performed when the acetyl-CoA:isopenicillin *N* acyltransferase (IAT), which catalyzes the last step in the penicillin biosynthesis was amplified from the *P. chrysogenum penDE* gene and expressed in *Hansenula polymorpha* (Lutz et al., 2005). However, most of the times the main obstacle is the large size (>40 kbp) of the DNA fragment that needs to be cloned, the effective activation/maturation of the expressed enzymes, and the toxicity of the produced compounds (Rutledge and Challis, 2015). Alternatively, fungi may be used as platform organism, as it was for instance demonstrated with the reconstruction of the citrinin gene cluster of *Monascus purpurea* in *A. oryzae* (Spraker and Keller, 2014). Likewise, the *in vivo* assembly of genetic elements has been successfully applied in *P. chrysogenum* through the overlapping of bi-partite fragments that reconstituted a functional *amdS* gene (marker), which eventually is integrated in the genome of this fungus proving the uncharacterized potential of *P. chrysogenum* as heterologous host (Polli et al., 2016). The potential of this approach follows a recent study employing *A. nidulans* as a host for the plasmid based expression of a diverse range of BGCs from other filamentous fungi (Clevenger et al., 2017).

The introduction of revolutionary new genetic tools, such as CRISPR/Cas9 offers more effective solutions to express specific BGCs. Such methods can contribute to product identification but also to the production of unique compounds by introduction of specific tailoring enzymes. These are the main strategies that are used for the activation of silent BGCs or for the modification/redirection of known biosynthetic pathways in order to increase NP diversification (Smanski et al., 2016). Specifically, this involves the expression of pathways from a plasmid in a suitable production host and a screen for product formation.

## Concluding Remarks

For many years *P. chrysogenum* has been used as one of the main industrial strains to produce penicillins (β-lactams). Its genome sequence revealed an unexplored potential of *P. chrysogenum* as a source of NPs (van den Berg et al., 2008). Despite the development of bioinformatics tools for genome mining of BGCs to identify novel molecules (Blin et al., 2017), the experimental validation of product structure and identity is still necessary. However, most of the secondary metabolite associated genes in *P. chrysogenum* are silent or poorly expressed. Given the urgent need for new molecules based on novel chemical scaffolds for the use in the medical and biotechnological fields (e.g., antibiotics, anti-cancer agents, antivirals, nutraceuticals, pigments, surfactants and many more), the use of organisms that have been genetically domesticated offers a promising target solution for NP discovery due to the availability of molecular tools for their genetic modification. Here, we have summarized the main approaches that have been applied for *P. chrysogenum* and other filamentous fungi to bioengineer secondary metabolite BGC pathways which have led to a greater understanding of the main obstacles to be overcome to use this fungus as a generic cell factory. We discussed the main characteristics of the building enzymes (PKS and NRPS) in filamentous fungi. Despite the apparent modular organization, the complexity of these mega enzymes and the inherent interactions between the various domains within their structures has not allowed a straight forward approach for the PKS/NRPS engineering (Thirlway et al., 2012; Zhang et al., 2013; Weissman, 2016). However, the combinatorial swapping strategy of structural elements such as recognition regions has increased the perspectives for designing *de novo* biosynthetic pathways. Further research needs to be focused around PKS/NRPS engineering in filamentous fungi to facilitate the rational design of biosynthetic enzymes to produce another generation of novel compounds. To mine the secondary metabolome of filamentous fungi, general methods such as manipulating cultivation conditions have been used that can also be implemented as a high-throughput strategy. Another avenue is interfering with the genetic regulatory systems, either through the manipulation of specific or global regulators. This strategy also revealed crosstalk between certain BGCs and an important role of chromatin remodeling in BGC expression. Because of its pleiotropic effect that leads to the activation or silencing of biosynthetic pathways, chromatin remodeling might be used as a more general strategy to explore the production of new metabolites in filamentous fungi (Guzmán-Chávez et al., 2018).

A further major advance is the development of genome-editing tools that allow for efficient genetic engineering of complex fungal cell factories (Jakoèiunas et al., 2016). In *P. chrysogenum*, improved methods for homologous recombination and CRISPR/Cas9 as genome editing tool now facilitates more advanced engineering of this fungus (Pohl et al., 2016, 2018). This is further stimulated by the development of a synthetic biology toolbox using promoters and terminators as building blocks and more complex regulatory devices to control the expression of genes. Importantly, the *in vivo* assembly of genetic elements in *P. chrysogenum* offers a promising tool to build entire pathways from scratch with reducing cloning efforts at minimal costs (Polli et al., 2016). In particular the low cost of DNA synthesis will allow rapid progress using such approaches as exemplified by a study using *A. nidulans* as a host where a diverse set of BGCs was expressed from an extra-chromosomal vector, the AMA plasmid (Clevenger et al., 2017). A further challenge is the generation of a platform strain in which endogenous BGCs have been removed to allow for more optimal carbon and nitrogen flow toward the production of the compounds of interest. Herein, the CRISPR/Cas9 based methods are instrumental (Pohl et al., 2016, 2018). Such a *Penicillium* platform might be used as a heterologous host to express a vast arsenal of BGCs from others filamentous fungi and represents a good alternative to yeast as expression host, an organism that does not naturally produce NRP and PK. The use of a such industrial strains to rapidly achieve the high level production of a novel metabolite has proven to be successful for pravastatin production (McLean et al., 2015) but a further step is to make use of secondary metabolite deficient industrial strains.

Despite the progress in genetic engineering and bioinformatics tools to identify BGCs, the main bottleneck to identify potentially interesting compounds has not yet been solved. Bioinformatic tools perform poorly in the prediction of the structures formed, and therefore future discovery programs will mostly dependent on high throughput methods to express foreign pathways and then use advanced metabolomics to identify the novel products. Since such approaches depend on high throughput, further efforts are needed to implement high throughput cloning methods to *P. chrysogenum* which will enable further studies to harness the enormous untapped source for NPs hidden in fungal (meta-)genomes.

## Author Contributions

FG-C and RZ wrote the manuscript. AD supervised, conceived, and designed the manuscript. RB co-supervised the manuscript.

## Conflict of Interest Statement

RB is an employee of DSM Biotechnology, Delft. The remaining authors declare that the research was conducted in the absence of any commercial or financial relationships that could be construed as a potential conflict of interest.
